# Atypical presentation of autoimmune hepatitis–primary sclerosing cholangitis overlap syndrome associated with hypereosinophilia: a case report and review of the literature

**DOI:** 10.1186/s13256-021-03086-9

**Published:** 2021-10-19

**Authors:** Behzad Hatami, Hamide Rahmani Seraji, Mohaddese fallahi

**Affiliations:** 1 Gastroenterology and Liver Diseases Research Center, Research Institute for Gastroenterology and Liver Diseases, Shahid Beheshti University of Medical Sciences, Tehran, Iran; 2 Department of Hematology and Oncology, Shahid Beheshti University of Medical Sciences, Tehran, Iran; 3 Department of Pathology, Shahid Beheshti University of Medical Sciences, Tehran, Iran; 4 Department of Hematology and Oncology, Shahid Beheshti University of Medical Sciences, Tehran, Iran

**Keywords:** Autoimmune hepatitis, Primary sclerosing cholangitis, Overlap syndrome, Hypereosinophilia

## Abstract

**Background:**

Autoimmune hepatitis–primary sclerosing cholangitis overlap syndrome is a form of autoimmune hepatitis with cholestatic features and is characterized by negative anti-mitochondrial antibody and cholangiographic changes on magnetic resonance cholangiopancreatography or endoscopic retrograde cholangiopancreatography. Peripheral blood hypereosinophilia in conjunction with autoimmune hepatitis–primary sclerosing cholangitis overlap syndrome has not been reported yet. Here we present a case of autoimmune hepatitis–primary sclerosing cholangitis overlap syndrome with hypereosinophilia.

**Case presentation:**

A 33-year-old Iranian man with the fatigue, jaundice, elevated liver enzymes and alkaline phosphatase, and hypereosinophilia was referred to our hospital. Viral and autoimmune hepatitis were excluded, and secondary workups for hypereosinophilia were all negative. Magnetic resonance cholangiopancreatography showed beaded appearance of intra- and extrahepatic biliary tree, and liver biopsy revealed interface hepatitis. Therefore, the diagnosis of autoimmune hepatitis–primary sclerosing cholangitis overlap syndrome was made, and prednisolone, azathioprine, and ursodeoxycholic acid was initiated. His jaundice and peripheral blood eosinophilia resolved after 2 weeks, and he became completely asymptomatic.

**Conclusion:**

Eosinophils might contribute to the clinical presentation and disease complications.

## Background

Autoimmune hepatitis–primary sclerosing cholangitis (AIH-PSC) overlap syndrome is a clinical, biochemical, immunological, and histological feature of AIH with cholangiographic abnormalities characteristic of PSC [1]. The frequency of the disease ranges from 1% to 6% [2] and is more common in males than females [1]. AIH or PSC concomitant with peripheral blood hypereosinophilia (HE) is a rare condition that has been described as case series [3–5], but to the best of our knowledge, peripheral blood hypereosinophilia in conjunction with AIH-PSC overlap syndrome has not been reported yet. Here we present a case of AIH-PSC overlap syndrome with hypereosinophilia.

## Case presentation

**Fig. 1:**
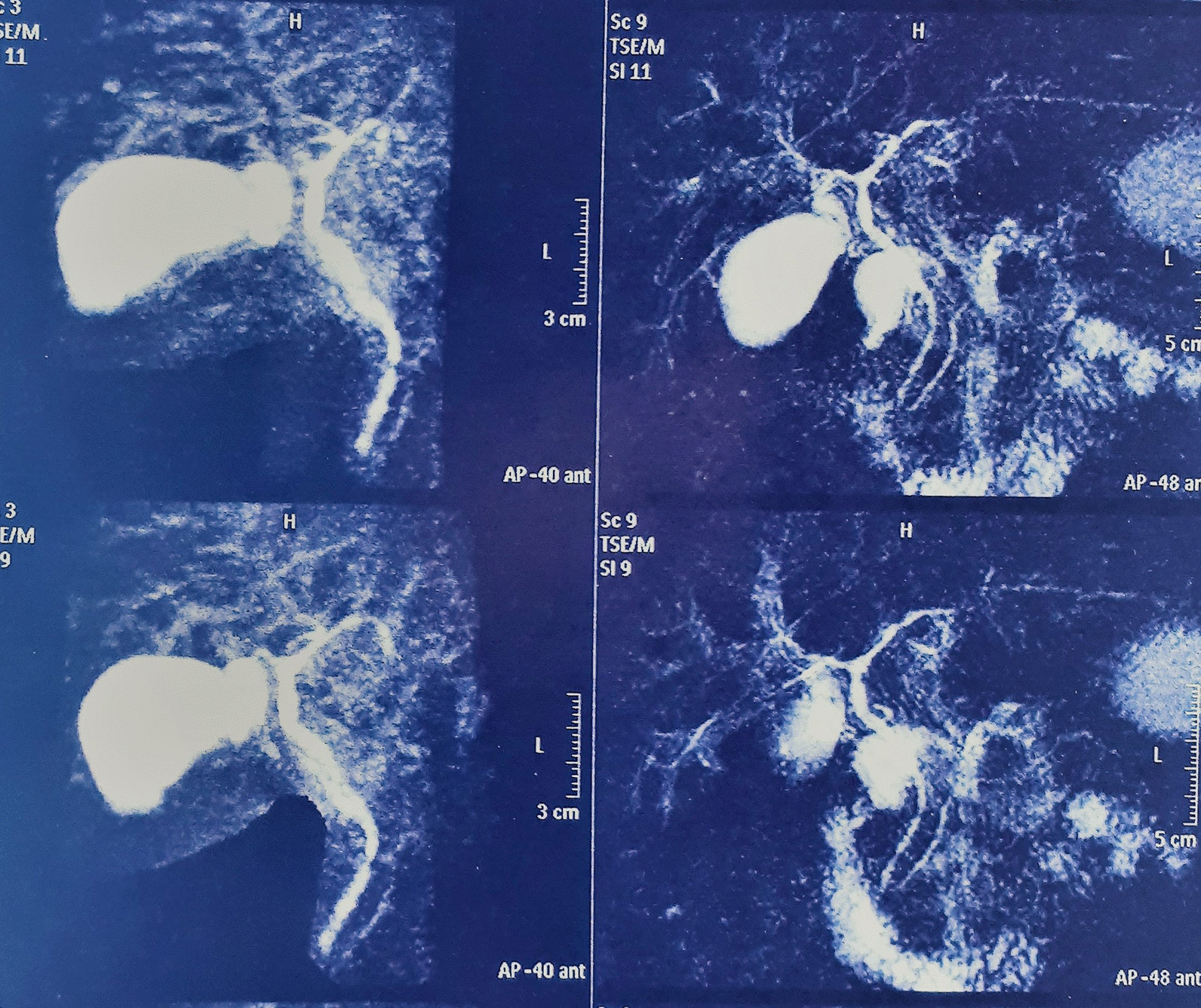
Magnetic resonance cholangiopancreatography (MRCP) findings. MRCP at diagnosis showed irregularity in the CHD, CBD, and intrahepatic bile ducts with multiple strictures

**Fig. 2:**
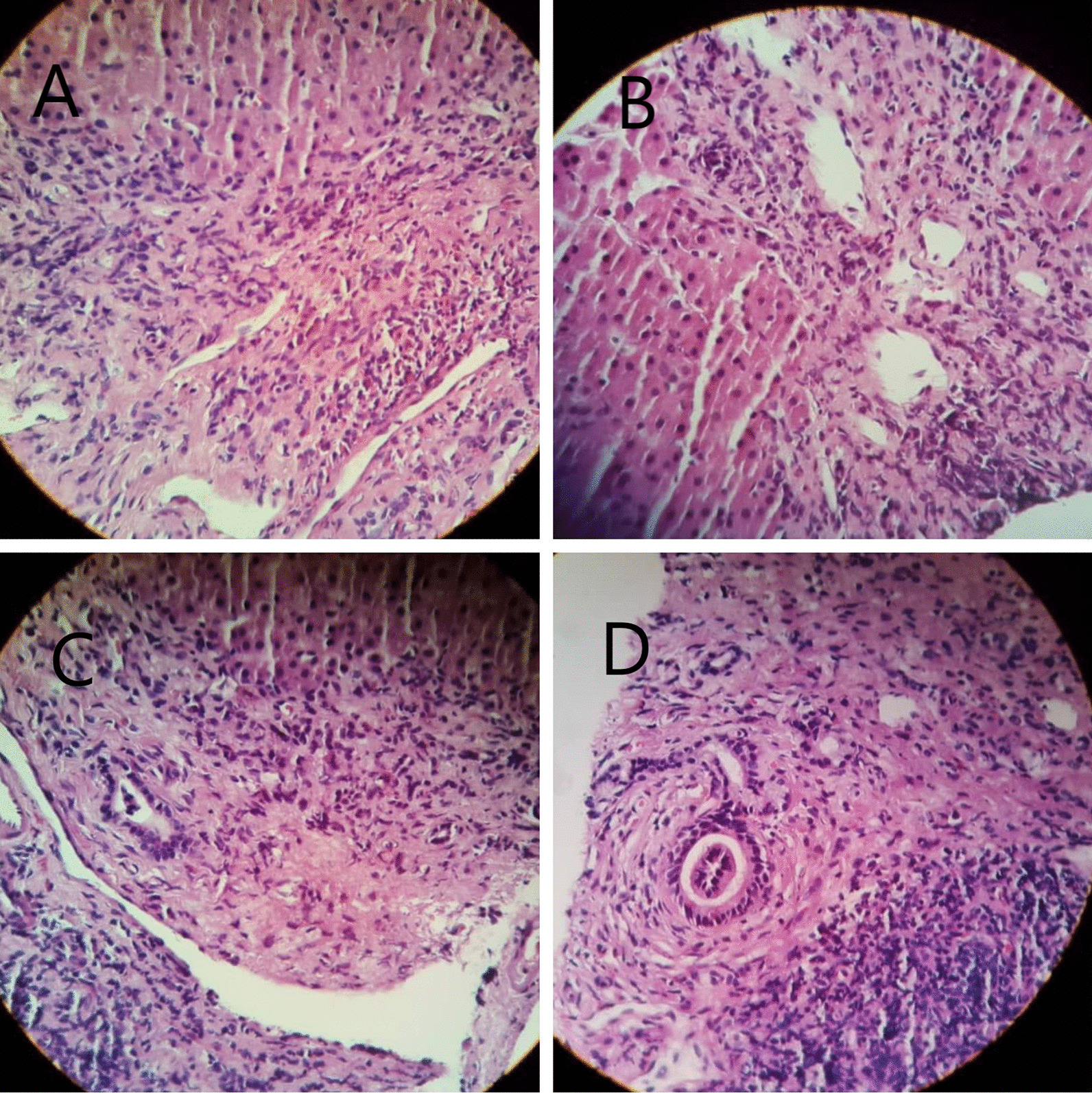
Liver biopsy revealing moderate infiltration of lymphocytes, plasma cells, and eosinophils in portal area leading to interface hepatitis (**A**–**C**). Eosinophil infiltration (**A**) and lymphocyte and plasma cell infiltration in portal area (**B**, **C**). Mild fibrosis around bile ducts in onion-skin-like pattern was noted, but cholestasis and steatosis were not seen (**D**)

A 33-year-old Iranian man was referred to our hospital because of fever, pruritus, jaundice, fatigue, dark urine, and pale stool for 3 months. He had no history of allergic disorder or recent travel. Medical history was unremarkable for gastrointestinal, liver, hematologic, or infectious disease. He had not used any medication. He was single and had no history of any high-risk sexual behavior. Family history was negative. On physical examination, sclera was icteric, and skin lesion, hepatosplenomegaly, lymphadenopathy, and ascites were not detected. The rest of the examinations were normal. In our hospital, laboratory data demonstrated a pattern of hypereosinophilia and hepatocellular liver injury: alanine transaminase (ALT) 710 IU/L (normal 10–40 IU/L), aspartate transaminase (AST) 389 IU/L (normal 5–40 IU/L), alkaline phosphatase (ALP) 677 IU/L (40 130 IU/L), gamma glutamyl transferase (GGT) 167 U/L (8–61 U/L), total bilirubin 3.45 mg/dl (0.3–1.2 mg/dl), direct bilirubin 2.79 mg/dl (0–0.3 mg/dl), prothrombin time (PT) 12.6 seconds, international normalized ratio (INR) 1.27 (0.8–1.2). The complete blood count included: white blood cells (WBC) 15,630/mm^3^, hemoglobin 15.5 g/dl, platelets 152,000/mm^3^, eosinophils 53%, with absolute eosinophil count of 8283/mm^3^. Serum IgG level was 2200 mg/dl (700–1400 mg/dl). Serum IgG4 level was normal, and serum protein electrophoresis and immune fixation revealed polyclonal gammopathy. Infectious diseases were excluded by negative results for viral markers [hepatitis B surface antigen and hepatitis C virus antibody, hepatitis A virus antibody (IgM), and anti-human immunodeficiency virus (HIV) antibody], serum antibodies to helminthic parasites, serologic test for *Fasciola hepatica*, and stool examinations for parasites and protozoa were all negative. Also, the workup for autoimmune hepatitis [antinuclear antibodies (ANA), smooth muscle antibody (SMA), anti-liver kidney microsomal type 1 (anti-LKM antibody 1) and anti-mitochondrial antibody (AMA)] was negative. Myeloproliferative diseases-related eosinophilia was excluded based on negative results for BCR-ABL P210 and P190 fusion transcript, JAK2 V617F mutation, Del [4] (q12q12)/FIP1L1PDGFRA, and PDGFRB/TEL. Bone marrow biopsy showed normal marrow hematopoiesis with increased morphologically normal eosinophils (account for 30% of all nucleated cells in bone marrow). Flow cytometry was normal as well. Abdominal ultrasonography and computed tomography scan of the abdomen and chest were unremarkable. Colonoscopy of the patient was normal. MRCP (Fig. 1) showed irregularity in the common hepatic duct (CHD) and common bile duct (CBD) walls with multiple narrowing. Also, fine irregularity in the intrahepatic bile ducts with multiple strictures, beaded appearance, and some prominent lymph nodes in porta hepatis measuring up to 32 × 13 mm size were seen. Liver biopsy was done for the patient and revealed moderate infiltration of lymphocytes, plasma cell, and eosinophils in the portal area leading to interface hepatitis and multiple focal and confluent necrosis. Bile duct proliferation was not seen. Mild fibrosis around bile ducts in onion-skin-like pattern was noted, but cholestasis and steatosis were not seen (Fig. 2).

**Fig. 3:**
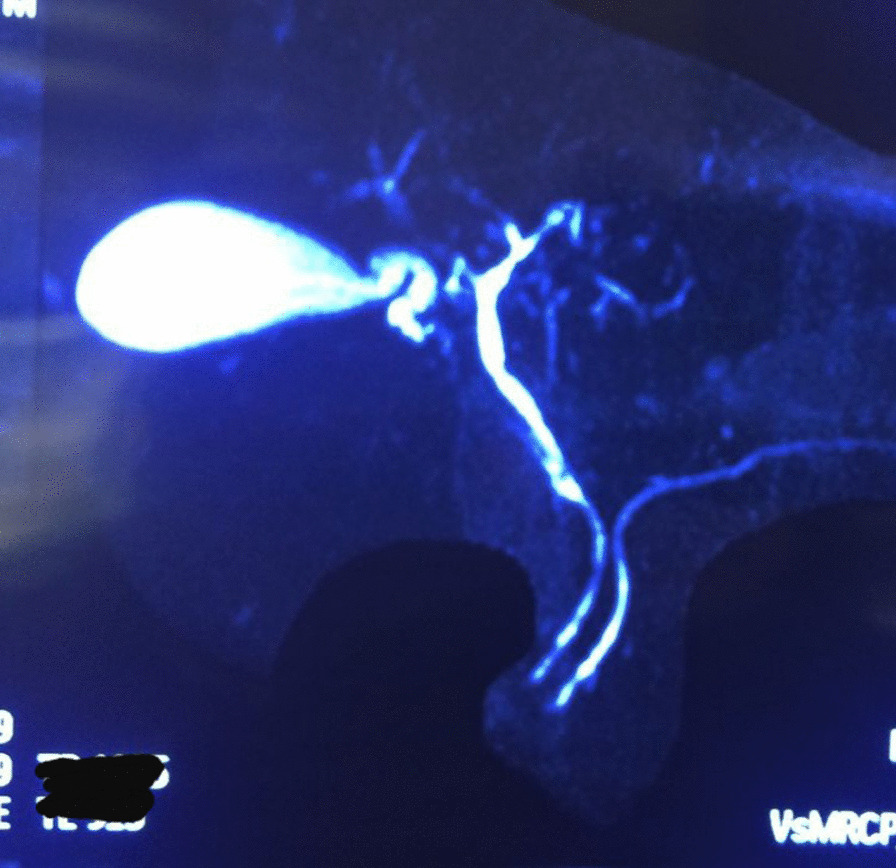
Second MRCP of the patient after 6 months

The diagnosis of AIH-PSC overlap syndrome was made, and prednisolone 30 mg/day, azathioprine 50 mg/day, and ursodeoxycholic acid (UDCA) 300 mg three times per day was commenced. Prednisolone was gradually tapered to 5 mg during 2 months. His jaundice and peripheral blood eosinophilia resolved after 2 weeks, and he became completely asymptomatic. Table 1 presents laboratory data of the patient at the time of diagnosis and 1, 6, and 12 months after initiation of treatment. MRCP of the patient after 6 months showed intrahepatic bile ducts, CHD, and CBD luminal irregularity without dilation (Fig. 3).

**Table 1:** Laboratory data of the patient at the time of diagnosis and 1, 6, and 12 months after initiation of treatment

Laboratory data	At diagnosis	1 month after treatment	6 months after treatment	12 months after diagnosis
Total bilirubin (mg/dl)	3.45	1.2	1.01	1.01
Direct bilirubin (mg/dl)	2.79	0.2	0.2	0.2
AST (IU/L)	710	35	30	30
ALT (IU/L)	389	20	25	20
ALP (IU/L)	677	100	67	65
GGT (U/L)	167	54	60	60
WBC (per mm^3^)	15,630	7000	5500	5000
Absolute eosinophil count (per mm^3^)	8283	150	100	125

## Discussion and conclusion

AIH/PSC overlap syndrome is a form of AIH with cholestatic features and is characterized by negative AMA and cholangiographic changes on MRCP or ERCP [6, 7]. Although eosinophilia is rarely reported in association with either AIH or PSC alone [8, 9], there has been no report of eosinophilia in association with AIH/PSC overlap syndrome, to the best of our knowledge. In our case, irrespective of noticeable peripheral blood eosinophilia, we made the diagnosis of AIH/PSC based on the mixed pattern of elevated liver enzymes, hypergammaglobulinemia, beaded appearance of intra- and extrahepatic biliary tree on MRCP, and marked interface hepatitis in liver histopathology. On the other hand, due to HE, hypereosinophilic syndrome with hepatic involvement was also suggested.

Hypereosinophilia (HE) in the peripheral blood is defined as an absolute eosinophil count (AEC) > 1.5 × 10/L (or > 1500 cells/μL) on two examinations separated in time by at least 1 month, and hypereosinophilic syndrome (HES) is defined by the association of HE with eosinophil-mediated organ damage and/or dysfunction [10] that is classified into three categories. Primary (or neoplastic) HES occurs in the setting of an underlying stem cell, myeloid, or eosinophilic neoplasm, and was excluded in our case according to laboratory data and bone marrow biopsy (BMB) findings as mentioned above. Secondary (or reactive) HES was excluded based on negative results for allergies or parasitic infections and finally idiopathic HES in which the underlying cause of HE remains unknown despite a thorough etiologic workup. The last type of HES cannot be excluded for our patient, although he had AIH-PSC overlap syndrome as an underlying disease, but there is no evidence to confirm AIH-PSC overlap syndrome as the underlying cause of HE. Kawamura *et al*. summarized reports of liver injury associated with hypereosinophilia in ten cases of active hepatitis with HES. Common findings on liver biopsy of the participants were portal inflammation and infiltration of eosinophils in this area [11], similar to findings of liver biopsy in our patient. On the other hand, the presence of an onion-skin pattern, which is the most specific histologic finding in PSC, goes against the diagnosis of HES. PSC with HE and eosinophilic cholangiopathy are two different entities; it is sometimes difficult to distinguish between them, and most clinical evidence about them has come from case reports [12]. Ichikawa *et al*. presented a case of HES with PSC according to bile duct changes on endoscopic retrograde cholangiopancreatography (ERCP) that was treated with prednisolone and UDCA. Dramatic response to prednisolone was observed, and he was completely asymptomatic after 2 months [13]. In cases reported by Grauer *et al*. [8] and Schoonbroodt *et al*. [9], improvement of the bile duct lesion in repeated ERCP after treatment was established as well. Such a dramatic response to corticosteroid may suggest that the bile duct involvement of these patients was related to eosinophilia. In our case, symptoms (fever, pruritus, jaundice, fatigue, dark urine, and pale stool), liver function tests, and HE quickly improved after initiation of treatment with prednisolone, azathioprine, and UDCA. This may suggest that HE is involved in the pathogenesis of the disease, although the existence of bile duct changes on follow-up MRCP contradicts this. In conclusion, we here reported a case of AIH-PSC overlap syndrome associated with HE in which eosinophils might contribute to the clinical presentation and disease complications.

## Data Availability

The datasets used during the current study are available from the corresponding author on reasonable request.

## Abbreviations

AIH-PSC: Autoimmune hepatitis–primary sclerosing cholangitis
HE: Hypereosinophilia
MRCP: Magnetic resonance cholangiopancreatography
CHD: Common hepatic duct
CBD: Common bile duct
